# Neighborhood built environment and physical activity of Japanese older adults: results from the Aichi Gerontological Evaluation Study (AGES)

**DOI:** 10.1186/1471-2458-11-657

**Published:** 2011-08-19

**Authors:** Tomoya Hanibuchi, Ichiro Kawachi, Tomoki Nakaya, Hiroshi Hirai, Katsunori Kondo

**Affiliations:** 1 Research Center for Disaster Mitigation of Urban Cultural Heritage, Ritsumeikan University, 58 Komatsubara Kitamachi, Kita-ku, Kyoto, Kyoto, 603-8341 Japan; 2 Department of Society, Human Development, and Health, Harvard School of Public Health, 677 Huntington Ave., Boston, MA, 02115 USA; 3 Department of Geography, Ritsumeikan University, 56-1 Tojiin-Kitamachi, Kita-ku, Kyoto, 603-8577 Japan; 4 Department of Civil Environmental Engineering, Faculty of Engineering, Iwate University, Morioka, Japan; 5 Center for Well-being and Society, Nihon Fukushi University, 5-22-35 Chiyoda, Naka-ku, Nagoya, 460-0012 Japan

## Abstract

**Background:**

Although many studies have reported the association between neighborhood built environment (BE) and physical activity (PA), less is known about the associations for older populations or in countries besides the US and Australia. The aim of this paper is to examine the associations for older adult populations in Japan.

**Methods:**

Our analyses were based on cross-sectional data from the Aichi Gerontological Evaluation Study (AGES), conducted in 2003. The respondents were older adults, aged 65 years or over (n = 9,414), from 8 municipalities across urban, suburban, and rural areas. The frequency of leisure time sports activity and total walking time were used as the outcome variables. Using geographic information systems (GIS), we measured residential density, street connectivity, number of local destinations, access to recreational spaces, and land slope of the respondents' neighborhoods, based on network distances with multiple radii (250 m, 500 m, 1,000 m). An ordinal logistic regression model was used to analyze the association between PA and BE measures.

**Results:**

Population density and presence of parks or green spaces had positive associations with the frequency of sports activity, regardless of the selected buffer zone. The analysis of total walking time, however, showed only a few associations.

**Conclusions:**

Our findings provide mixed support for the association between PA and the characteristics of BE measures, previously used in Western settings. Some characteristics of the neighborhood built environment may facilitate leisure time sports activity, but not increase the total walking time for Japanese older adults.

## Background

Physical activity (PA) has been reported to have many health benefits including reduced risk of mortality [1,2] and the prevention of chronic diseases, such as cardiovascular disease, diabetes, cancer, hypertension, obesity, or depression [3]. However, a large part of the population does not regularly engage in exercise. For example, less than one third of the Japanese population (32.2% of men and 27.0% of women) regularly engage in exercise; thirty minutes or more of exercise two or more times per week, for more than a year [4]. In this regard, exploring factors that are associated with increased levels of physical activity is important for public health research.

In studies of the health impacts of neighborhood environments, the association between the built environment (BE) and physical activity is of central importance [5]. PA has been reported to be related to residential density, street connectivity, and land use mix [6-8]. Although many empirical studies have analyzed the associations using perceived measures [9-11], objective measures [12-15], or both [16-19], the findings continue to be heterogeneous (e.g., no association, or associations in the opposite direction) [20,21]. This may be explained by variations in the environmental measures, study populations, or geographical settings, in which the respondents reside.

Age (adolescent, middle aged, or older adults) is an important source of the between-study heterogeneity. Compared to the studies of adolescents and adults, few studies have explored the association between the BE and the PA of older adults [14,22]. Only recently, especially in the late 2000s, have researchers begun to analyze the association with a variety of objective and/or perceived measures of BE (e.g., residential density, land use mix, street connectivity, access to local destinations, walking/cycling facilities, etc.) and different types of PA (e.g., total PA, recreational PA, recreational walking, transportation walking, etc.). In a systematic review of 31 articles concerning the relationship between BE and PA in older adults, Van Cauwenberg et al. [23] concluded that the results were inconsistent, though most of the studied environmental characteristics were reported to be unrelated to PA. The authors pointed out that this might reflect some methodological issues within this developing field, such as the measurement of PA and environment. For example, Nagel et al. [14] found no association between any of the variables of objectively measured BEs and the likelihood of engaging in walking, based on the samples of community-dwelling older adults in Portland, Oregon. However, amongst those reporting some degree of walking activity, the average time spent walking was associated with some variables of BE; amount of automobile traffic and number of commercial establishments.

Furthermore, the broader social and cultural context may be important in studies of the environment and PA. Although many studies have been conducted, especially in the US, Europe, and Australia [23], the association between BE and PA may not be clearly generalizable to other societies. For example, a study of an elderly population from Latin America (Bogotá) recently showed the negative associations between street connectivity and walking for at least 60 minutes, which, according to the authors, differs from most of the evidence gathered from studies in Europe and the US [24]. Generally speaking, the spatial forms of residence, transportation infrastructure, and retail or business locations may all vary according to the given country, region and cultural context. Thus, research is needed to explore the association between the BE and PA outside the US, Europe, and Australia [7,23,25], especially in Asian countries, where a dearth of studies have been conducted.

Based on the above mentioned challenges, the aim of this paper is to fill the gaps in the literature by examining the association between neighborhood BEs and PA of older adults in Japan.

## Methods

### Data

Our analyses were based on the cross-sectional data of the Aichi Gerontological Evaluation Study (AGES), conducted in 2003 [26]. We conducted a mail survey with a random sample of functionally independent, community-dwelling people aged 65 years and over (i.e., who were not eligible for public, long-term nursing care) in 15 municipalities from 3 prefectures in Japan. According to the availability of geocoded data, the present study involved 11,876 respondents from 8 municipalities (response rate = 48.7%) in the Chita Peninsula region. The Chita Peninsula region is adjacent to Nagoya City, which is the center of the third largest metropolitan area in Japan. The study area consisted of eight municipalities that included urban/suburban areas (northern part of the Chita Peninsula) and rural areas (southern part). The study protocol and informed consent procedure were approved by the Ethics Committee in Research of Human Subjects at Nihon Fukushi University.

### Outcome

Frequency of leisure time sports activity and total walking time were the two outcome variables used.

#### Leisure time sports activity

The questions for frequency of leisure time activities, including sports activity, were included in the questionnaire [27]. Respondents were asked "*Do you engage in any leisure activities at the moment?*" with possible responses of "*Yes*" or "*No*". Those who answered "*Yes*" were then asked about the frequency of leisure activities for each of the eight types (sports activities, cultural activities, musical activities, creative activities, horticulture etc., watching TV etc., travelling etc., and gambling etc.). Sports activity was included as one of these types, with the description of: "*sports activities (e.g., ground golf; "gateball" [Japanese croquet]; walking; jogging; physical exercises)*". Possible responses were the following six categories: *I don't engage in any sports activities, I engage in the activity several times a year, once or twice a month, once a week, twice or three times a week, and almost everyday*. Those who did not engage in any leisure activities (from the first question) or who did not engage in sports activities (from the second question) were collapsed into a single category.

#### Walking

We also inquired about the total walking time per day. Respondents were asked "*How long do you walk a day on average?*" with the following four response categories: *Less than 30 minutes, 30 to 60 minutes, 60 to 90 minutes, and more than 90 minutes*. The purpose of walking (e.g., for transportation or for leisure) was not identified in this question. A previous study reported the validity of a single-item questionnaire on walking among the Japanese population, using pedometer counts as the reference standard [28].

### Exposure

#### Definition of neighborhoods

For neighborhood BEs, we measured residential density, street connectivity, number of local destinations, accessibility to recreational facilities, and land slope. Neighborhoods were defined by constructing a buffer zone around each respondent's home, based on a street network. Compared to the commonly used multilevel structure (where individuals are nested in a larger geographic unit), the GIS approach allows for neighborhood environments to be defined for each resident level. Since the relevant size of a neighborhood could vary according to the age group or other settings, the use of multiple geographic scales was helpful in this regard [6]. Given that our study population consisted of older adults, we considered a radial distance of 250 m as indicating the most accessible space, in addition to 500 m and 1,000 m (roughly a quarter mile and a half mile).

We used ArcGIS 9.3 for all spatial calculations. The "CSV address matching service" (provided by the Center for Spatial Information Science, The University of Tokyo) was used for geocoding procedures. The accuracy of geocoding was at the *Gaiku *(city block) level; reference points were located at about 50 m intervals.

#### Residential density

Population density was used as an indicator of residential density. Nevertheless, similar to the suggestion by Owens et al. [29], regarding the US census data, the Japanese census unit (*Chou-Chou-Aza-tou*) is unsuitable for measuring neighborhood population density, especially in suburban and rural areas, since the unit tends to be large and includes many non-habitable areas. To address this problem, we identified developed areas as those with buildings or settlements at 50 m interval points, based on the 1:25,000 Topographic Map in Japan [30]. Next, each point was weighted by the population of the census unit (as of 2005). For example, if the population was 500, and 20 points were identified in a certain census unit, we assigned a population of 25 to each point. We then aggregated the population of the neighborhood, based on the points within the network buffer. With this method, we could exclude non-developed areas (e.g., rivers, ponds, or mountains) and some land use for non-residential purposes (farms or industrial districts) from the calculation.

#### Street connectivity

The number of intersections (at least three-way) was used as an index of street connectivity. We also counted the number of dead-end streets, as possibly representing lower street connectivity. The source of information was the Digital Map 2500 (Spatial Data Framework), published by The Geospatial Information Authority of Japan, which provides basic spatial data on streets, public spaces, natural environments, and administrative boundaries, as of 2002.

#### Number of local destinations

The number of destinations was used as a measure for the land use mix. Considering previous studies and the Japanese context, we chose 17 common destinations: bank, bookstore, cafe, clothing store, community center, convenience store, dentist, electrical appliance shop, fast-food store, hairdressing salon, hospital, laundry, library, municipal office, pharmacy, post office, and supermarket. The data was collected from the Yellow Pages, a phone number database, in August 2010, and geocoded.

#### Recreational facilities

The presence or absence of parks or green spaces and schools was measured as the accessibility to recreational facilities. Parks or green spaces also included open spaces, athletic grounds, and ball parks. Schools were included in our analysis because some schools open their grounds to the public. This information was obtained from the Digital Map 2500 (Spatial Data Framework).

#### Land slope

Average land slope of neighborhoods was measured for the neighborhood environment, though this could be considered a feature of the physical or natural environment, rather than the BE. Elevation data (as of 2001) was obtained from the Digital Map 50 m Grid (Elevation), from The Geospatial Information Authority of Japan.

### Covariates

Considering possible confounding factors from the respondents' demographic, socioeconomic, and health status, age (65-69, 70-74, 75-79, 80-84, ≥85 years); gender (male, female); marital status (married, divorced/widowed, never married); educational attainment (<6, 6-9, 10-12, ≥13 years of schooling); household equivalized income (<1 million, 1-2 million, 2-3 million, 3-4 million, and ≥4 million yen); having paid work (Yes, No); self-rated health (SRH; fair and poor were collapsed into Poor); 15-item Geriatric Depression Scale (GDS; 10 points or more = High depressive symptoms); and instrumental activities of daily living (IADL; 4 points or less = Low IADL) by TMIG-IC [31] were used as the control variables.

### Statistical analysis

Based on the ordinal scale of our two outcome variables, we performed an ordinal logistic regression analysis. BE measures were included as continuous (population density, number of intersections, number of dead-ends, number of destinations, and land slope) or dummy variables (parks or green spaces, and schools), though the variables that were categorized into quartiles (Lowest, Low, High, and Highest) were also considered in order to examine the non-linear association. First, each BE measure was separately included in the regression model due to the high correlations among them. We also considered mutually adjusted models including the variables that were shown to be associated with PA in the separated models. A regression analysis was also performed for the sample after stratifying the data by gender, location (North vs. South), and years of residence in the municipality (<50 years vs. ≥50 years), in order to explore whether the associations between BE and PA were seen in each population subgroup.

The analyses were restricted to respondents who provided complete information on age and gender, and who were successfully geocoded. For other control variables, we created a "missing" category for missing data. Respondents from two isolated islands were excluded from our analyses, because our measurements were based on network distance, and the evaluation of accessibility for these respondents would be difficult. Finally, we used 9,414 older adults for the analyses, though the number of samples for the regression varied due to missing values for the outcome variables.

## Results

Basic characteristics of the respondents are shown in Table 1 and characteristics of the BEs in neighborhoods are shown in Table 2. More than half respondents did not engage in sports activity. However, among those who did engage in sports activity, many people were more likely to engage in it frequently. As for walking time, although approximately one third of the respondents answered they walk less than 30 minutes a day, the responses were more evenly distributed than the frequency of sports activity. Table 3 represents the correlation coefficient between BE measures. Some combinations showed a high correlation, especially at the radial distance of 1,000 m. For example, population density was positively correlated with the number of intersections (r = 0.66) and the number of destinations (r = 0.74).

**Table 1:** Characteristics of the respondents

	n	%		n	%
Overall	9414	100.0	Equivalized income		
Sports activity			<1 million yen	937	10.0
No sports activities	5227	55.5	1-2 million yen	2123	22.6
Several times a year	70	.7	2-3 million yen	2161	23.0
Once or twice a month	230	2.4	3-4 million yen	1451	15.4
Once a week	676	7.2	≥4 million yen	929	9.9
Twice or three times a week	1176	12.5	Missing	1813	19.3
Almost everyday	1495	15.9	Having paid work		
Missing	540	5.7	Yes	2272	24.1
Walking time/day			No	6970	74.0
Less than 30 minutes	2936	31.2	Missing	172	1.8
30 to 60 minutes	3074	32.7	SRH		
60 to 90 minutes	1183	12.6	Good	6591	70.0
More than 90 minutes	1104	11.7	Poor	2585	27.5
Missing	1117	11.9	Missing	238	2.5
Age			GDS		
65-69	3386	36.0	Low depressive symptoms	7401	78.6
70-74	2765	29.4	High depressive symptoms	565	6.0
75-79	1886	20.0	Missing	1448	15.4
80-84	938	10.0	IADL		
≥85	439	4.7	High IADL	7178	76.2
Gender			Low IADL	1837	19.5
Male	4519	48.0	Missing	399	4.2
Female	4895	52.0	Location		
Marital status			North	3856	41.0
Married	6759	71.8	South	5558	59.0
Divorced/widowed	2321	24.7	Years of residence		
Never married	127	1.3	<50 years	4819	51.2
Missing	207	2.2	≥50 years	4138	44.0
Educational attainment			Missing	457	4.9
<6	430	4.6			
6-9	5242	55.7			
10-12	2711	28.8			
≥13	903	9.6			
Missing	128	1.4			

**Table 2:** Characteristics of neighborhoods of respondents

	n	Mean	SD	Min	Max
(r = 250 m)					
Population density (per hectare)	9414	35.3	18.3	0.0	140.8
No. of intersections	9414	22.4	10.3	0.0	69.0
No. of dead-ends	9414	2.8	2.5	0.0	18.0
No. of destinations	9414	2.6	3.2	0.0	33.0
Parks or green spaces	9414	0.1	0.3	0.0	1.0
Schools	9414	0.1	0.3	0.0	1.0
Land slope	9414	2.9	2.2	0.0	14.5
(r = 500 m)					
Population density (per hectare)	9414	34.0	16.5	0.0	108.5
No. of intersections	9414	70.6	27.3	2.0	176.0
No. of dead-ends	9414	7.8	6.0	0.0	39.0
No. of destinations	9414	8.2	7.7	0.0	57.0
Parks or green spaces	9414	0.1	0.3	0.0	1.0
Schools	9414	0.3	0.5	0.0	1.0
Land slope	9414	2.7	1.6	0.1	12.0
(r = 1000 m)					
Population density (per hectare)	9414	25.2	13.4	1.3	91.7
No. of intersections	9414	252.1	91.4	16.0	552.0
No. of dead-ends	9414	39.7	23.0	1.0	108.0
No. of destinations	9414	29.6	21.6	0.0	117.0
Parks or green spaces	9414	0.2	0.4	0.0	1.0
Schools	9414	0.6	0.5	0.0	1.0
Land slope	9414	3.1	2.0	0.2	12.7

**Table 3:** Correlation coefficient between BE measures

		a)	b)	c)	d)	e)	f)	g)
(r = 250 m)								
a)	Population density	1.00						
b)	No. of intersections	0.31	1.00					
c)	No. of dead-ends	-0.07	-0.01	1.00				
d)	No. of destinations	0.23	0.32	0.02	1.00			
e)	Parks or green spaces	0.39	0.16	-0.13	0.08	1.00		
f)	Schools	0.04	0.10	0.03	0.10	0.02	1.00	
g)	Land slope	-0.29	-0.24	0.01	-0.18	-0.08	0.04	1.00
(r = 500 m)								
a)	Population density	1.00						
b)	No. of intersections	0.42	1.00					
c)	No. of dead-ends	-0.01	0.17	1.00				
d)	No. of destinations	0.44	0.44	0.07	1.00			
e)	Parks or green spaces	0.43	0.29	-0.22	0.18	1.00		
f)	Schools	0.07	0.19	-0.01	0.17	0.09	1.00	
g)	Land slope	-0.27	-0.38	-0.07	-0.32	-0.08	-0.01	1.00
(r = 1000 m)								
a)	Population density	1.00						
b)	No. of intersections	0.66	1.00					
c)	No. of dead-ends	0.15	0.35	1.00				
d)	No. of destinations	0.74	0.58	0.23	1.00			
e)	Parks or green spaces	0.43	0.42	-0.20	0.25	1.00		
f)	Schools	0.04	0.12	-0.01	0.03	0.25	1.00	
g)	Land slope	-0.45	-0.59	-0.26	-0.43	-0.19	0.08	1.00

Table 4 shows the results of the ordinal logistic regression analysis for leisure time sports activity. Population density was related to increased sports activity at radial distances of 250 m (OR = 1.004, 95%CI = 1.001-1.006), 500 m (OR = 1.004, 95%CI = 1.002-1.007), and 1,000 m (OR = 1.005, 95%CI = 1.002-1.008) for the neighborhood. The presence of parks or green spaces also showed a consistent association with sports activity at 250 m (OR = 1.258, 95%CI = 1.082-1.462), 500 m (OR = 1.152, 95%CI = 1.021-1.300), and 1,000 m radius (OR = 1.162, 95%CI = 1.056-1.280). The number of dead-ends was inversely related to sports activity, at radial distances of 500 m (OR = 0.992, 95%CI = 0.985-0.999), while the number of intersections had a positive association only at a radial distance of 1,000 m (OR = 1.001, 95%CI = 1.000-1.001). The presence of schools was not associated with sports activity measures. Land slope were negatively related to sports activity at 250 m (OR = 0.961, 95%CI = 0.941-0.981), 500 m (OR = 0.957, 95%CI = 0.931-0.983), and 1000 m radius (OR = 0.944, 95%CI = 0.923-0.967).

**Table 4:** Associations between frequency of sports activity and each of the BEs by ordinal logistic regression analysis

		r = 250 m		r = 500 m		r = 1000 m	
Independent variables ^a^		OR^b^	95%CI	OR^b^	95%CI	OR^b^	95%CI
Population density		1.004	(1.001, 1.006)	1.004	(1.002, 1.007)	1.005	(1.002, 1.008)
No. of intersections		1.001	(0.997, 1.006)	1.001	(0.999, 1.002)	1.001	(1.000, 1.001)
No. of dead-ends		0.991	(0.975, 1.008)	0.992	(0.985, 0.999)	1.000	(0.998, 1.002)
No. of destinations		1.002	(0.989, 1.015)	1.000	(0.994, 1.005)	1.001	(0.999, 1.003)
Parks or green spaces		1.258	(1.082, 1.462)	1.152	(1.021, 1.300)	1.162	(1.056, 1.280)
Schools		0.967	(0.852, 1.097)	1.008	(0.919, 1.106)	0.992	(0.909, 1.082)
Land slope		0.961	(0.941, 0.981)	0.957	(0.931, 0.983)	0.944	(0.923, 0.967)
(Quartiles)							
Population density (Ref. Lowest)	Low	1.017	(0.900, 1.150)	0.899	(0.796, 1.015)	1.074	(0.950, 1.215)
	High	1.067	(0.945, 1.205)	0.971	(0.861, 1.096)	1.160	(1.027, 1.310)
	Highest	1.186	(1.054, 1.335)	1.147	(1.021, 1.29)	1.200	(1.066, 1.351)
No. of intersections (Ref. Lowest)	Low	1.052	(0.934, 1.184)	1.075	(0.952, 1.212)	1.233	(1.088, 1.397)
	High	1.063	(0.943, 1.198)	0.982	(0.869, 1.108)	1.138	(1.004, 1.290)
	Highest	1.034	(0.919, 1.163)	1.089	(0.966, 1.228)	1.191	(1.051, 1.350)
No. of dead-ends (Ref. Lowest)	Low	0.880	(0.778, 0.994)	0.896	(0.800, 1.002)	0.929	(0.824, 1.047)
	High	0.852	(0.762, 0.952)	0.926	(0.823, 1.041)	0.990	(0.879, 1.116)
	Highest	0.954	(0.852, 1.069)	0.884	(0.787, 0.992)	0.999	(0.888, 1.123)
No. of destinations (Ref. Lowest)	Low	1.095	(0.984, 1.219)	1.096	(0.972, 1.236)	1.185	(1.049, 1.338)
	High	1.097	(0.969, 1.243)	1.101	(0.982, 1.233)	1.069	(0.948, 1.206)
	Highest	1.042	(0.919, 1.181)	0.980	(0.872, 1.100)	1.161	(1.033, 1.306)
Land slope (Ref. Lowest)	Low	1.129	(1.001, 1.273)	1.026	(0.910, 1.156)	1.059	(0.941, 1.193)
	High	1.115	(0.988, 1.258)	1.088	(0.965, 1.227)	1.053	(0.934, 1.187)
	Highest	0.948	(0.837, 1.073)	0.933	(0.825, 1.057)	0.840	(0.740, 0.953)

^a ^Each independent variable was included separately.

^b ^Age, gender, marital status, educational attainment, equivalized income, having paid work, SRH, GDS, and IADL were adjusted.

When looking at the results using variables that were categorized into quartiles, some non-linear associations were observed. For the number of dead-ends at 250 m radius, Low (OR = 0.880, 95%CI = 0.778-0.994) and High (OR = 0.852, 95%CI = 0.762-0.952) showed differences compared to the reference category (Lowest), but Highest (OR = 0.954, 95%CI = 0.852-1.069) did not. Although no linear relation was observed, when using the categorized variable, the number of destinations was associated with sports activity at a radial distance of 1,000 m (Low: OR = 1.185, 95%CI = 1.049-1.338, Highest: OR = 1.161, 95%CI = 1.033-1.306). Mixed results were observed for land slope; a negative association was observed at a radial distance of 1,000 m (Highest: OR = 0.840, 95%CI = 0.740-0.953), while a positive association was observed at a radial distance of 250 m (Low: OR = 1.129, 95%CI = 1.001-1.273).

Table 5 represents the results of mutually adjusted models including the variables that were shown to be associated with sports activity in the separated models at each buffer radius. Associations remained for two out of three BE variables at 250 m radius, three out of four at 500 m radius, and two out of four at 1,000 m radius.

**Table 5:** Associations between frequency of sports activity and BE (mutually adjusted)

	r = 250 m		r = 500 m		r = 1000 m	
Independent variables ^a^	OR^b^	95%CI	OR^b^	95%CI	OR^b^	95%CI
Population density	1.002	(0.999, 1.004)	1.003	(1.000, 1.006)	1.002	(0.997, 1.006)
No. of intersections					1.000	(0.999, 1.000)
No. of dead-ends			0.992	(0.985, 0.999)		
Parks or green spaces	1.186	(1.007, 1.397)	1.036	(0.902, 1.190)	1.118	(1.003, 1.247)
Land slope	0.966	(0.945, 0.987)	0.964	(0.938, 0.992)	0.947	(0.921, 0.975)

^a ^Independent variables were included simultaneously.

^b ^Age, gender, marital status, educational attainment, equivalized income, having paid work, SRH, GDS, and IADL were adjusted.

Table 6 shows the results of regression analysis for total walking time. Only a few associations were observed. The land slope showed a consistent positive association with walking time at 250 m (OR = 1.037, 95%CI = 1.018-1.056), 500 m (OR = 1.048, 95%CI = 1.023-1.074), and 1,000 m radius (OR = 1.036, 95%CI = 1.015-1.058), suggesting that the respondents living in areas with steeper slopes tended to report longer times for walking per day. When using variables that were categorized into quartiles, in addition to the land slope, the number of intersections at a radial distance of 500 m (Low: OR = 0.876, 95%CI = 0.782-0.981, Highest: OR = 0.891, 95%CI = 0.796-0.998) and the number of destinations at a radial distance of 1,000 m (Low: OR = 0.879, 95%CI = 0.784-0.985) were negatively associated with walking time.

**Table 6:** Associations between total walking time and each of the BEs by ordinal logistic regression analysis

		r = 250 m		r = 500 m		r = 1000 m	
Independent variables ^a^		OR^b^	95%CI	OR^b^	95%CI	OR^b^	95%CI
Population density		1.000	(0.998, 1.002)	0.999	(0.997, 1.002)	0.999	(0.996, 1.002)
No. of intersections		0.997	(0.993, 1.001)	0.999	(0.998, 1.001)	1.000	(0.999, 1.000)
No. of dead-ends		0.997	(0.981, 1.012)	0.997	(0.991, 1.004)	0.999	(0.997, 1.001)
No. of destinations		0.998	(0.986, 1.010)	0.996	(0.990, 1.001)	0.999	(0.997, 1.001)
Parks or green spaces		1.045	(0.902, 1.211)	1.057	(0.940, 1.188)	1.019	(0.929, 1.117)
Schools		1.078	(0.958, 1.214)	1.063	(0.974, 1.160)	1.085	(0.999, 1.178)
Land slope		1.037	(1.018, 1.056)	1.048	(1.023, 1.074)	1.036	(1.015, 1.058)
(Quartiles)							
Population density (Ref. Lowest)	Low	1.088	(0.973, 1.218)	0.971	(0.868, 1.086)	0.998	(0.892, 1.117)
	High	0.919	(0.820, 1.030)	0.900	(0.804, 1.008)	0.911	(0.813, 1.020)
	Highest	1.042	(0.933, 1.165)	0.970	(0.868, 1.084)	0.937	(0.838, 1.048)
No. of intersections (Ref. Lowest)	Low	0.941	(0.843, 1.051)	0.876	(0.782, 0.981)	0.992	(0.884, 1.113)
	High	0.926	(0.827, 1.037)	0.959	(0.856, 1.074)	0.914	(0.814, 1.027)
	Highest	0.937	(0.839, 1.046)	0.891	(0.796, 0.998)	0.998	(0.888, 1.121)
No. of dead-ends (Ref. Lowest)	Low	0.977	(0.871, 1.095)	1.031	(0.928, 1.145)	1.073	(0.960, 1.200)
	High	0.962	(0.867, 1.069)	0.930	(0.832, 1.041)	0.996	(0.890, 1.114)
	Highest	0.926	(0.832, 1.032)	0.937	(0.840, 1.046)	0.980	(0.877, 1.096)
No. of destinations (Ref. Lowest)	Low	0.930	(0.841, 1.029)	0.926	(0.827, 1.038)	0.879	(0.784, 0.985)
	High	1.011	(0.899, 1.136)	1.007	(0.905, 1.121)	1.035	(0.926, 1.156)
	Highest	0.977	(0.869, 1.098)	0.935	(0.838, 1.042)	0.942	(0.843, 1.052)
Land slope (Ref. Lowest)	Low	1.039	(0.927, 1.165)	1.025	(0.915, 1.149)	1.061	(0.947, 1.189)
	High	1.025	(0.914, 1.149)	1.053	(0.939, 1.180)	1.153	(1.029, 1.292)
	Highest	1.178	(1.049, 1.322)	1.189	(1.059, 1.334)	1.154	(1.027, 1.297)

^a ^Each independent variable was included separately.

^b ^Age, gender, marital status, educational attainment, equivalized income, having paid work, SRH, GDS, and IADL were adjusted.

The regression analysis was performed using subgroups stratified by gender, location (North vs. South Chita Peninsula), and years of residence (<50 years vs. ≥50 years). The results were shown in Table 7 (sports activity) and Table 8 (walking time). Regarding sports activity, the association between the BE and PA was clearly apparent among male respondents. Associations were observed in 10 out of the 21 models (seven BE measures and three buffer radii) for the male group, while only one was observed among the female respondents. In seven out of the 10 models, interactions between gender and BE were observed (results not shown). When the analysis was stratified by location, even though associations were seen in both North and South, only a few were observed in North. For example, parks or green spaces were not associated with sports activity in the North subgroup. Among respondents who had resided longer in the municipality (≥50 years), associations were observed in eight models, while only one was seen among respondents who had resided for a shorter time in the municipality (<50 years). For example, associations between population density and sports activity were only seen among the residents residing for 50 years or more (at three radii). Interactions between years of residence and BE were observed in three of the models.

**Table 7:** Associations between frequency of sports activity and the BE stratified by gender, location, and years of residence

	r = 250 m		r = 500 m		r = 1000 m	
Independent variables ^a^	OR^b^	95%CI	OR^b^	95%CI	OR^b^	95%CI
Male						
Population density	1.005	(1.002, 1.009)	1.007	(1.003, 1.010)	1.008	(1.003, 1.012)
No. of intersections	1.003	(0.997, 1.008)	1.001	(0.999, 1.003)	1.001	(1.000, 1.002)
No. of dead-ends	0.999	(0.976, 1.022)	0.993	(0.984, 1.003)	1.000	(0.998, 1.003)
No. of destinations	1.009	(0.991, 1.028)	0.997	(0.989, 1.005)	1.002	(0.999, 1.004)
Parks or green spaces	1.336	(1.089, 1.640)	1.253	(1.064, 1.476)	1.233	(1.081, 1.407)
Schools	0.854	(0.711, 1.024)	0.964	(0.846, 1.098)	0.972	(0.862, 1.096)
Land slope	0.943	(0.915, 0.972)	0.945	(0.909, 0.982)	0.924	(0.893, 0.956)
Female						
Population density	1.001	(0.998, 1.005)	1.002	(0.998, 1.006)	1.003	(0.998, 1.007)
No. of intersections	1.000	(0.994, 1.006)	1.000	(0.998, 1.003)	1.000	(1.000, 1.001)
No. of dead-ends	0.982	(0.959, 1.006)	0.990	(0.980, 1.000)	0.999	(0.997, 1.002)
No. of destinations	0.993	(0.974, 1.012)	1.002	(0.995, 1.010)	1.001	(0.998, 1.003)
Parks or green spaces	1.172	(0.937, 1.464)	1.033	(0.862, 1.238)	1.083	(0.939, 1.249)
Schools	1.092	(0.916, 1.301)	1.060	(0.928, 1.210)	1.019	(0.898, 1.157)
Land slope	0.979	(0.952, 1.008)	0.971	(0.935, 1.009)	0.964	(0.934, 0.995)
North						
Population density	1.001	(0.998, 1.004)	1.003	(0.999, 1.006)	1.001	(0.997, 1.006)
No. of intersections	1.007	(1.001, 1.013)	1.001	(0.998, 1.003)	1.000	(0.999, 1.001)
No. of dead-ends	0.987	(0.957, 1.019)	0.994	(0.979, 1.009)	0.998	(0.995, 1.002)
No. of destinations	1.017	(0.998, 1.035)	1.004	(0.996, 1.011)	1.000	(0.998, 1.003)
Parks or green spaces	1.138	(0.970, 1.336)	1.024	(0.893, 1.174)	1.007	(0.884, 1.147)
Schools	0.975	(0.809, 1.175)	0.999	(0.874, 1.143)	0.920	(0.783, 1.081)
Land slope	0.954	(0.907, 1.003)	0.968	(0.911, 1.029)	1.041	(0.965, 1.123)
South						
Population density	1.004	(1.000, 1.008)	1.005	(1.000, 1.009)	1.008	(1.003, 1.012)
No. of intersections	0.995	(0.989, 1.001)	1.000	(0.998, 1.002)	1.001	(1.000, 1.001)
No. of dead-ends	1.004	(0.984, 1.025)	0.998	(0.989, 1.007)	1.002	(1.000, 1.004)
No. of destinations	0.989	(0.970, 1.008)	0.996	(0.988, 1.004)	1.001	(0.999, 1.004)
Parks or green spaces						
Schools	0.951	(0.799, 1.132)	0.977	(0.857, 1.115)	0.941	(0.840, 1.055)
Land slope	0.963	(0.941, 0.986)	0.953	(0.924, 0.983)	0.943	(0.919, 0.967)
< 50 years						
Population density	1.001	(0.998, 1.003)	1.002	(0.998, 1.005)	1.001	(0.997, 1.005)
No. of intersections	1.002	(0.996, 1.007)	1.000	(0.998, 1.002)	1.000	(1.000, 1.001)
No. of dead-ends	0.990	(0.968, 1.012)	0.995	(0.985, 1.004)	0.999	(0.996, 1.001)
No. of destinations	1.007	(0.988, 1.026)	0.999	(0.992, 1.006)	0.998	(0.996, 1.001)
Parks or green spaces	1.198	(1.011, 1.420)	1.086	(0.942, 1.251)	1.051	(0.931, 1.185)
Schools	0.990	(0.829, 1.181)	1.029	(0.905, 1.170)	0.975	(0.869, 1.094)
Land slope	0.975	(0.942, 1.009)	0.979	(0.938, 1.022)	0.970	(0.932, 1.010)
≥ 50 years						
Population density	1.005	(1.001, 1.009)	1.005	(1.000, 1.010)	1.007	(1.001, 1.012)
No. of intersections	0.999	(0.992, 1.005)	1.000	(0.998, 1.003)	1.001	(1.000, 1.001)
No. of dead-ends	1.001	(0.975, 1.028)	0.993	(0.982, 1.004)	1.002	(0.999, 1.004)
No. of destinations	1.003	(0.984, 1.023)	1.002	(0.994, 1.010)	1.005	(1.002, 1.008)
Parks or green spaces	1.034	(0.708, 1.509)	1.065	(0.821, 1.381)	1.217	(1.022, 1.450)
Schools	0.976	(0.808, 1.179)	1.029	(0.892, 1.185)	1.041	(0.905, 1.198)
Land slope	0.968	(0.942, 0.995)	0.961	(0.926, 0.997)	0.954	(0.926, 0.984)

^a ^Each independent variable was included separately.

^b ^Age, gender, marital status, educational attainment, equivalized income, having paid work, SRH, GDS, and IADL were adjusted.

**Table 8:** Associations between total walking time and the BE stratified by gender, location, and years of residence

	r = 250 m		r = 500 m		r = 1000 m	
Independent variables ^a^	OR^b^	95%CI	OR^b^	95%CI	OR^b^	95%CI
Male						
Population density	1.002	(0.999, 1.005)	1.001	(0.998, 1.005)	1.002	(0.998, 1.006)
No. of intersections	0.999	(0.993, 1.004)	1.000	(0.998, 1.002)	1.000	(1.000, 1.001)
No. of dead-ends	1.000	(0.978, 1.022)	0.998	(0.988, 1.007)	0.999	(0.997, 1.002)
No. of destinations	0.993	(0.976, 1.012)	0.997	(0.989, 1.004)	1.000	(0.997, 1.002)
Parks or green spaces	1.133	(0.925, 1.386)	1.157	(0.985, 1.360)	1.085	(0.954, 1.233)
Schools	1.122	(0.945, 1.331)	1.003	(0.886, 1.136)	1.053	(0.938, 1.181)
Land slope	1.028	(1.000, 1.056)	1.038	(1.002, 1.074)	1.021	(0.991, 1.053)
Female						
Population density	0.998	(0.995, 1.001)	0.997	(0.994, 1.001)	0.996	(0.991, 1.000)
No. of intersections	0.995	(0.990, 1.001)	0.998	(0.996, 1.000)	1.000	(0.999, 1.000)
No. of dead-ends	0.994	(0.972, 1.016)	0.997	(0.988, 1.007)	0.999	(0.997, 1.002)
No. of destinations	1.003	(0.986, 1.020)	0.995	(0.988, 1.002)	0.998	(0.996, 1.001)
Parks or green spaces	0.953	(0.770, 1.181)	0.951	(0.802, 1.128)	0.948	(0.829, 1.083)
Schools	1.043	(0.885, 1.229)	1.130	(0.999, 1.278)	1.124	(0.998, 1.265)
Land slope	1.045	(1.019, 1.072)	1.058	(1.022, 1.094)	1.048	(1.019, 1.078)
North						
Population density	1.000	(0.997, 1.003)	1.000	(0.997, 1.004)	1.002	(0.998, 1.006)
No. of intersections	0.999	(0.993, 1.005)	0.999	(0.997, 1.001)	1.000	(0.999, 1.001)
No. of dead-ends	0.989	(0.960, 1.019)	0.999	(0.985, 1.014)	1.002	(0.998, 1.005)
No. of destinations	1.006	(0.988, 1.024)	0.999	(0.992, 1.006)	1.001	(0.998, 1.004)
Parks or green spaces	0.990	(0.846, 1.159)	1.003	(0.878, 1.145)	0.931	(0.821, 1.057)
Schools	1.078	(0.899, 1.293)	0.994	(0.872, 1.132)	1.028	(0.878, 1.204)
Land slope	0.997	(0.950, 1.047)	1.004	(0.947, 1.065)	1.019	(0.948, 1.096)
South						
Population density	0.998	(0.994, 1.001)	0.996	(0.992, 1.000)	0.994	(0.989, 0.998)
No. of intersections	0.995	(0.989, 1.000)	0.999	(0.997, 1.001)	1.000	(0.999, 1.000)
No. of dead-ends	1.005	(0.986, 1.025)	1.000	(0.992, 1.008)	0.999	(0.997, 1.001)
No. of destinations	0.992	(0.975, 1.009)	0.992	(0.985, 1.000)	0.997	(0.995, 1.000)
Parks or green spaces						
Schools	1.084	(0.926, 1.267)	1.112	(0.987, 1.253)	1.081	(0.974, 1.201)
Land slope	1.043	(1.022, 1.065)	1.056	(1.028, 1.085)	1.042	(1.019, 1.066)
< 50 years						
Population density	1.002	(0.999, 1.004)	1.001	(0.998, 1.005)	1.001	(0.997, 1.005)
No. of intersections	1.001	(0.996, 1.007)	1.000	(0.998, 1.002)	1.000	(1.000, 1.001)
No. of dead-ends	0.987	(0.966, 1.008)	0.993	(0.984, 1.002)	0.999	(0.996, 1.001)
No. of destinations	1.006	(0.988, 1.025)	0.996	(0.989, 1.004)	0.999	(0.997, 1.002)
Parks or green spaces	1.049	(0.886, 1.243)	1.130	(0.982, 1.300)	1.091	(0.969, 1.229)
Schools	1.098	(0.922, 1.307)	1.029	(0.907, 1.168)	1.084	(0.968, 1.214)
Land slope	1.019	(0.986, 1.054)	1.034	(0.992, 1.077)	1.026	(0.988, 1.065)
≥ 50 years						
Population density	0.996	(0.993, 1.000)	0.996	(0.992, 1.001)	0.997	(0.992, 1.002)
No. of intersections	0.992	(0.986, 0.997)	0.998	(0.996, 1.000)	1.000	(0.999, 1.001)
No. of dead-ends	1.006	(0.982, 1.030)	1.001	(0.991, 1.011)	1.000	(0.997, 1.002)
No. of destinations	0.992	(0.975, 1.009)	0.995	(0.987, 1.002)	0.999	(0.996, 1.002)
Parks or green spaces	1.082	(0.770, 1.520)	0.925	(0.728, 1.175)	0.982	(0.835, 1.155)
Schools	1.095	(0.926, 1.295)	1.092	(0.961, 1.240)	1.075	(0.947, 1.219)
Land slope	1.044	(1.020, 1.069)	1.056	(1.023, 1.090)	1.035	(1.008, 1.062)

^a ^Each independent variable was included separately.

^b ^Age, gender, marital status, educational attainment, equivalized income, having paid work, SRH, GDS, and IADL were adjusted.

In terms of walking time, a clear difference was seen between North and South. Among the respondents who resided in the South, associations were detected in eight models, and the direction of the associations were negative for population density, number of intersections, and number of destinations, and positive for land slope. Out of the eight models, three models showed interactions between location and BE measures.

## Discussion

The present study revealed that, in Japan, some neighborhood BEs were associated with the PA levels of older residents. For example, population density and the presence of parks or green spaces were associated with increased sports activity, regardless of the buffer zone selected. Land slope was also consistently associated with sports activity in the expected direction (i.e., negative association), but only when it was used as continuous variable. The number of destinations was not linearly related to the frequency of sports activity, while using the variable, categorized into quartiles, yielded some positive associations. In the mutually adjusted models, some BE variables were not associated with sports activity, suggesting that not all the BE variables have independent effects on sports activity, or, that it can be difficult to disentangle the effects of BEs due to their similar spatial distribution. On the other hand, the results of the analysis for total walking time showed only a few associations, and most of them were in an unexpected direction. Therefore, our findings provide mixed support for the association between PA and the characteristics of BEs, previously used in Western settings.

Our study also showed mixed results based on the stratified samples. In particular, the unexpected direction of the associations between the BE and walking time (i.e., negative associations for population density, the number of intersections, and number of destinations, and positive associations for land slope) in Southern Chita Peninsula deserves comment. As a possible explanation for this finding, the respondents who were engaged in farming or forestry may have considered their daily work routines to be walking time. Southern Chita is more rural than northern Chita. Additionally, respondents who do not drive a car (most likely females) may have had to walk longer in areas with less public transportation.

As for gender differences, associations with sports activity were mostly seen among the male respondents. In particular, population density, parks or green spaces, and land slope showed associations with sports activity at any of the buffer radii. This may reflect gender differences in the context of sports activity. For older men, games or sports events (e.g., ground golf or gateball [Japanese croquet]) are more likely to be accessible and preferred, while daily physical activities (e.g., walking or jogging), that can be performed alone or with a few people, may be more popular among older women. Neighborhood BEs, such as population density or availability of parks, may play a role especially for such games or events. Regarding years of residence, many associations were observed in the subgroup of residents residing for 50 years or more. This may suggest that the results are not necessarily attributable to reverse causation brought about by differences in residential preference.

The present study has some advantages. Our analysis was based on the respondents of Japanese older adults. Although PA levels have been reported to be related to neighborhood BEs, less is known about these associations for older adults or in countries besides the US, Europe, and Australia [14,22,23]. Some studies have recently reported associations between BEs and PA in Japan; however, they were mainly based on perceived measures [9,10,32]. A few studies have analyzed a limited set of BE characteristics using GIS techniques [33], or have focused on small samples from a narrow study area [34]. In contrast, we measured various BEs using GIS, based on network distances with multiple radii. Particularly, a method for calculating population density, based on a combination of topographic maps and census data, may have contributed to a more accurate measurement and the detection of its association with sports activity.

In addition, using samples from a variety of regions, covering eight municipalities with urban, suburban, and rural areas, was another advantage in the current study. Van Cauwenberg et al. [23] pointed out that the low number of positive relationships in previous studies of older adults could be due to the limited range of environmental variation in the study areas. Since broader regional variations in the BE may occur between urban and rural areas, analysing respondents from a variety of regions might have allowed us to detect some associations between BE and PA. In our stratified analysis by location (North vs. South), results similar to the entire sample were obtained for the South subgroup, while only a few associations were seen for the North strata. For example, parks or green spaces were not associated with sports activity when the analysis was limited within the North strata. Including samples from the South, the region which has less access to parks or green spaces (no parks or green spaces were located within 1,000 m from the respondents' home in the South region), may have contributed to the increasing regional variation and the detection of the association with sports activity.

Using multiple radii for buffer zones was also considered to be an advantage of this study, though the main results were basically consistent between all radii. However, as for street connectivity, the number of dead-ends was associated with sports activity at 250 m and 500 m, while the number of intersections showed an association at 1,000 m radius. Many dead-ends in immediate neighborhoods may indicate that the residents lived in a much less connected area. In the stratified analysis, there were also some different results for the selected buffer radii. For example, associations were only seen at 250 m radius in the North subgroup. Although this is not very clear from our results, an appropriate radius could vary by population, region, or types of BE. Therefore, using multiple radii would be useful, at least until an appropriate radius is established, and further study is needed to explore a better definition for neighborhood in many localities.

The present study also has some limitations. First, self-reported measured PA was a limitation, as they might have been misreported, and/or recognition of "leisure time" and "sports activity" might differ between each respondent. Our outcome variables were not based on well-established measures, such as the International Physical Activity Questionnaire (IPAQ). For example, we could not distinguish between walking for transport vs. walking for PA. Since specific environments may affect specific physical activities, the variety of physical activities should be considered, such as the purpose of walking (e.g., for daily errands, for leisure, or for commuting to work) [10]. In our study, leisure time sports activity was more specific in its purpose than total walking time, which could explain the different associations with the BE.

Another limitation of our study was its cross-sectional design, which prevents inferences being made about causality from the observed associations. Residents are likely to reside in different neighborhoods based partly on their preferences for PA. For example, those inclined to be regularly active may choose to live in areas that offer a variety of features (access to parks, sidewalks) that are conducive to PA. Recent studies have begun to tease out the effects of residential preferences, which may confound the associations between BE characteristics and PA. For example, Frank et al. [35] found that the low preference for living in a walkable neighborhood was indeed associated with less walking (for both transport and leisure). Importantly, within the strata of residential preferences, the objective BE independently predicted walking behavior. In other words, even among residents expressing a high preference for living in a highly walkable neighborhood, low walkability (objectively assessed by GIS) was associated with less walking.

Finally, some have suggested that the social environment influence PA [36], and that walkable BEs may increase social capital [37,38]. Thus, the neighborhood social capital is often considered as the causal pathway from the BE to PA [8,39]. Nevertheless, since the social environment may interact with the BE, it could be both a mediator and a confounder in the association between BE and PA. These complex causal relationships among the environmental features need to be further elucidated in future studies.

## Conclusions

The aim of this paper was to examine the association between the characteristics of neighborhood BEs and PA levels of older adults in Japan. Our findings provide mixed support for the association. Some characteristics of the neighborhood BE (e.g., population density and presence of parks or green spaces) may facilitate leisure time sports activity, but not increase the total walking time for Japanese older adults.

## Competing interests

The authors declare that they have no competing interests.

## Authors' contributions

TH conceived of the study, performed the statistical analysis, and drafted the manuscript. IK participated in the design of the study and helped to draft the manuscript. TN assisted in the statistical analysis and contributed to the interpretation of the results. HH and KK contributed to the data acquisition, interpretation of the results, and revision of the manuscript. All authors read and approved the final manuscript.

## Pre-publication history

The pre-publication history for this paper can be accessed here:

http://www.biomedcentral.com/1471-2458/11/657/prepub
